# Myeloid sarcoma as EM blast crisis in BCR-ABL1 e6a2+ CML with myelofibrosis and TB: a case report

**DOI:** 10.1515/biol-2025-1369

**Published:** 2026-08-31

**Authors:** Chunling Gu, Rui Lu, Yixin Kang, Wen Chen, Yongqing Zhang, Sainan Yang

**Affiliations:** Graduate School, Hebei North University, Zhangjiakou, Hebei Province, 075000, China; The Eighth Medical Center, Chinese PLA General Hospital, Beijing, 100089, China; Department of Pathology, The Eighth Medical Center, Chinese People’s Liberation Army (PLA) General Hospital, Beijing, China; Department of Hematology, The Eighth Medical Center of Chinese PLA General Hospital, Beijing, China

**Keywords:** chronic myeloid leukemia, BCR-ABL1 e6a2, myeloid sarcoma, myelofibrosis, tuberculosis, PET-CT

## Abstract

BCR-ABL1 e6a2-positive chronic myeloid leukemia (CML) is a rare molecular subtype of CML, accounting for less than 1 % of CML cases. It was shown to be associated with aggressive clinical behavior and a tendency toward disease progression. Here, we report a 56-year-old man who presented with pancytopenia, cervical lymphadenopathy, and severe myelofibrosis, without the typical leukocytosis observed in classic CML. Concurrent active pulmonary tuberculosis further complicated the diagnostic process. Hyperuricemia and elevated levels of lactate dehydrogenase (LDH) suggested a high cell-turnover state, and cervical lymph node biopsy confirmed myeloid sarcoma. Cytogenetic analysis revealed t(9;22)(q34;q11.2) with trisomy 8. Real-time quantitative polymerase chain reaction (RT-qPCR) identified the rare BCR-ABL1 e6a2 transcript. The patient received second- and third-generation tyrosine kinase inhibitor (TKI)-based therapy combined with chemotherapy. An individualized rifamycin-sparing anti-tuberculosis regimen was also prescribed. The patient achieved complete remission with marked regression of myelofibrosis. This case suggests that e6a2-positive CML may present with atypical features, including pancytopenia, severe myelofibrosis, and extramedullary blast crisis. In patients with concurrent tuberculosis, timely tissue biopsy and molecular testing are essential to accelerate diagnosis.

## 1 Introduction

CML is a myeloproliferative neoplasm driven by the t(9;22)(q34;q11.2) translocation, which results in the formation of the Philadelphia (Ph) chromosome [1]. This translocation forms the BCR-ABL1 fusion gene, encoding a tyrosine kinase with persistent activity [2]. More than 95 % of patients with CML express the classic p210 fusion protein (e13a2 or e14a2 transcript) [3], 4]. In contrast, the e6a2 transcript formed by the fusion of BCR exon 6 and ABL1 exon 2 is a rare variant, with an incidence of less than 1 % among patients with CML worldwide [5], [6], [7].

Compared with the classic p210 BCR-ABL1, the e6a2 fusion originates from an earlier breakpoint within BCR and may alter the regulatory domains involved in BCR-ABL1 signaling [8]. Previous studies have linked this rare transcript to aggressive clinical behavior, resistance to standard treatment, clonal evolution, and a tendency toward extramedullary involvement. Classic CML typically presents with marked leukocytosis, granulocytic hyperplasia, and splenomegaly [9], 10]. Here, we report a challenging patient with e6a2-positive CML with extramedullary blast crisis who initially presented with severe pancytopenia, marked myelofibrosis, and cervical lymphadenopathy. The coexistence of active pulmonary tuberculosis further obscured the diagnosis and increased the risk of misattributing lymphadenopathy and systemic symptoms to infection alone.

## 2 Case presentation

### 2.1 Clinical course and key milestones

The patient, a 56-year-old male, had a history of type 2 diabetes. He was recently diagnosed with active pulmonary tuberculosis (TB) and admitted to the Hematology Department due to progressive fatigue. The patient had a history of pulmonary tuberculosis, which was diagnosed several years before the last presentation. For pulmonary tuberculosis, the patient had completed a six-month course of anti-tuberculosis treatment. Approximately two months before the current admission, chest computed tomography (CT) at a local hospital revealed volume loss and consolidation in the right upper lobe, multiple cavities with air bronchograms, and bilateral pulmonary nodules. Subsequently, a sputum smear test was conducted, which returned positive for acid-fast bacilli; thus, the anti-tuberculosis regimen was re-initiated. After the initiation and subsequent modification of anti-tuberculosis treatment, repeated sputum smears and molecular tests for mycobacteria became negative. Thus, the diagnosis of active pulmonary tuberculosis was supported based on the patient’s history, typical cavitary upper-lobe lesions on chest imaging, and positive sputum smear.

**Figure 1:**
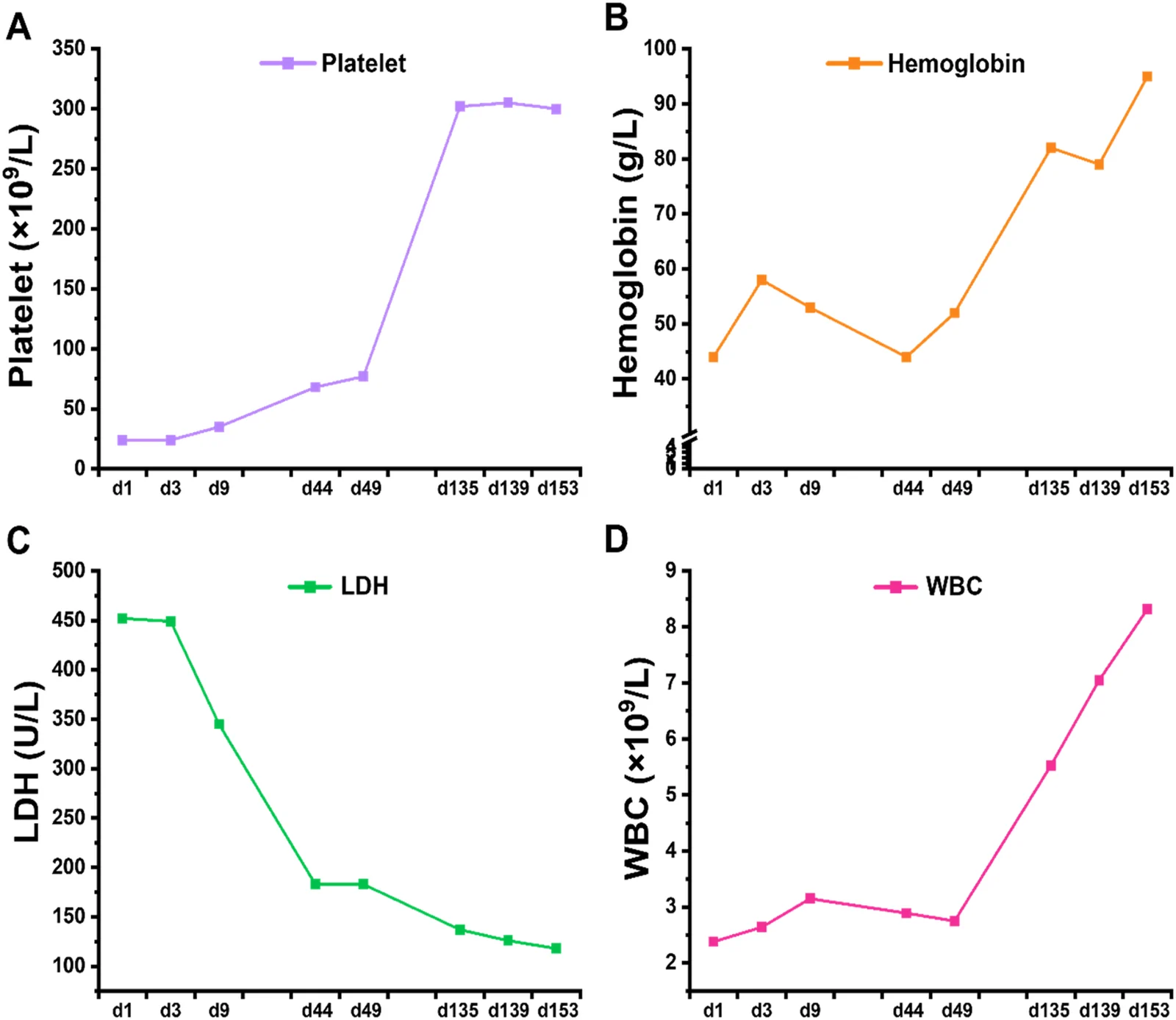
Trends of various indicators. (A–D) Changes in PLT count, Hb level, LDH level, and WBC: In the patient from diagnosis to treatment.

On admission, the patient had a dry cough and emaciation, but no obvious fever, sore throat, abdominal pain, or diarrhea. In addition, his general mental status and appetite were normal. Physical examination revealed multiple enlarged lymph nodes in the right neck, with the largest having a diameter of approximately 2 cm. The nodes were firm and non-tender. Initial peripheral blood (PB) analysis showed severe pancytopenia, with a white blood cell (WBC) count of 2.89 × 10^9^/L, a hemoglobin (Hb) level of 44 g/L, and a platelet (PLT) count of 77 × 10^9^/L. Peripheral blood smear revealed immature granulocytic and erythroid cells, teardrop-shaped red blood cells, and left-shifted granulocytes. Biochemical tests revealed significantly elevated serum levels of uric acid (up to 722.0 μmol/L) and increased serum levels of LDH, which were considerably inconsistent with the reduced peripheral WBC count. Based on the clinical course of the patient, including baseline severe pancytopenia, abnormal biochemical markers, and key events during subsequent treatment, a summary of the trajectory of biochemical indicators is presented in Figure 1.

**Informed consent:** Informed consent was obtained from all individuals included in this study, or their legal guardians or wards.

**Ethical approval:** The research related to human use has been complied with all the relevant national regulations, institutional policies and in accordance with the tenets of the Helsinki Declaration, and has been approved by the Ethics Committee of Chinese PLA General Hospital, Qinglongqiao Street, Haidian District, Beijing 100091, China (Approval No. S-2026-006-01).

### 2.2 Imaging assessment (PET-CT)

**Figure 2:**
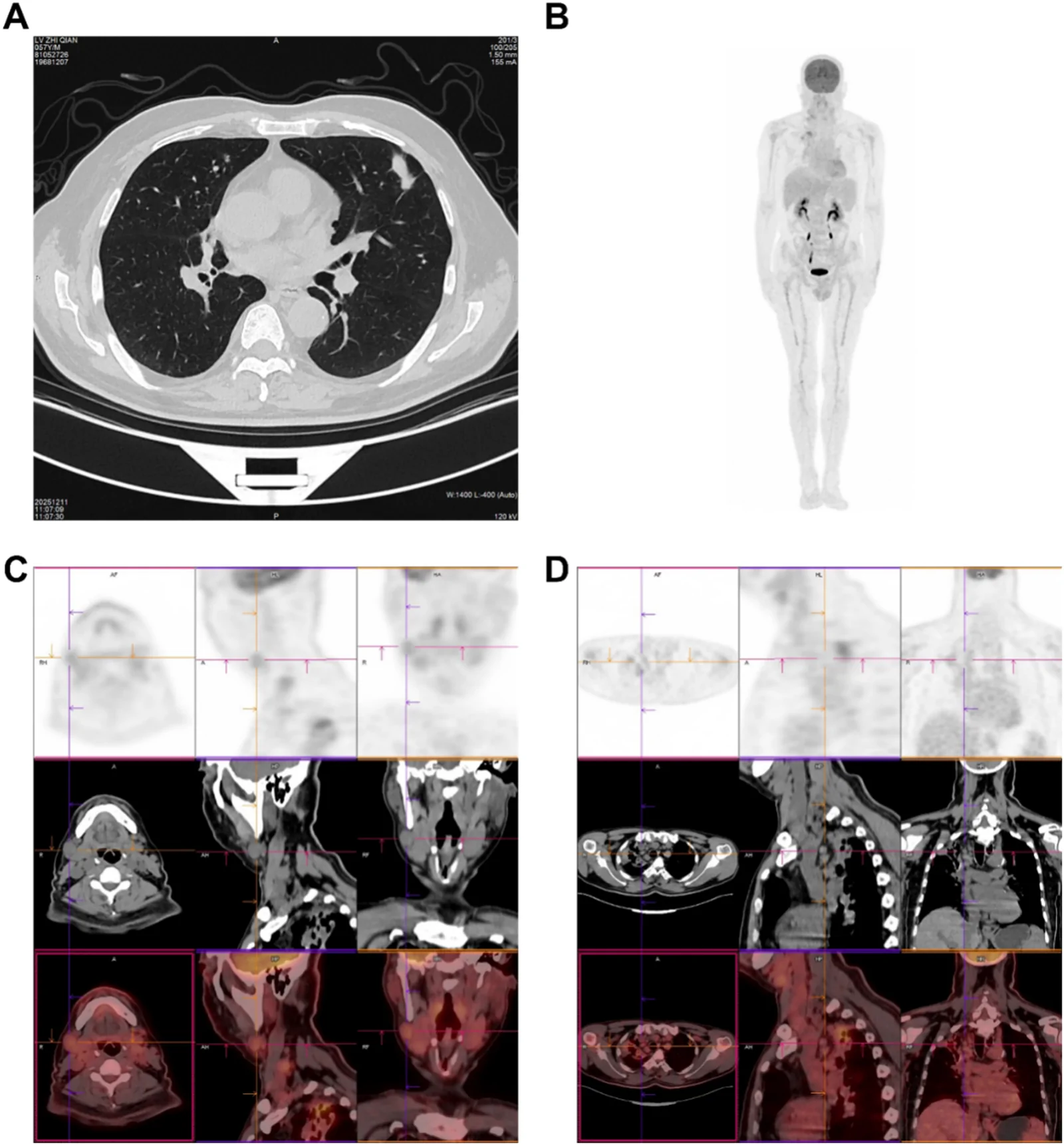
Imaging findings. (A) Multiple cystic and nodular opacities in both lungs, with air-fluid levels observed within the bronchi and cavities in the right upper lobe, (B–D) diffusely increased FDG uptake in the systemic bone marrow, and multiple enlarged lymph nodes with abnormally increased FDG uptake in both sides of the neck, supraclavicular regions, and the mediastinum.

The patient underwent whole-body 18F-FDG PET-CT to assess the extent of lymph node enlargement and concurrent active tuberculosis (Figure 2). PET-CT revealed FDG-avid cervical, supraclavicular, and mediastinal lymphadenopathy, as well as FDG-avid lesions in the upper lobes of both lungs. In CT images, cavitary and nodular pulmonary lesions were detected predominantly in the upper lobes. Combined with the initially positive sputum smear, these findings support active pulmonary tuberculosis. Although FDG uptake was not specific for malignancy and overlapped with tuberculosis-induced inflammation, the distribution pattern of lymphadenopathy and biochemical evidence of high cellular turnover, including elevated levels of uric acid and LDH, necessitated tissue biopsy. The axial bone marrow also showed altered metabolic activity, which was consistent with severe bone marrow fibrosis.

### 2.3 Pathological examination and molecular diagnosis

**Figure 3:**
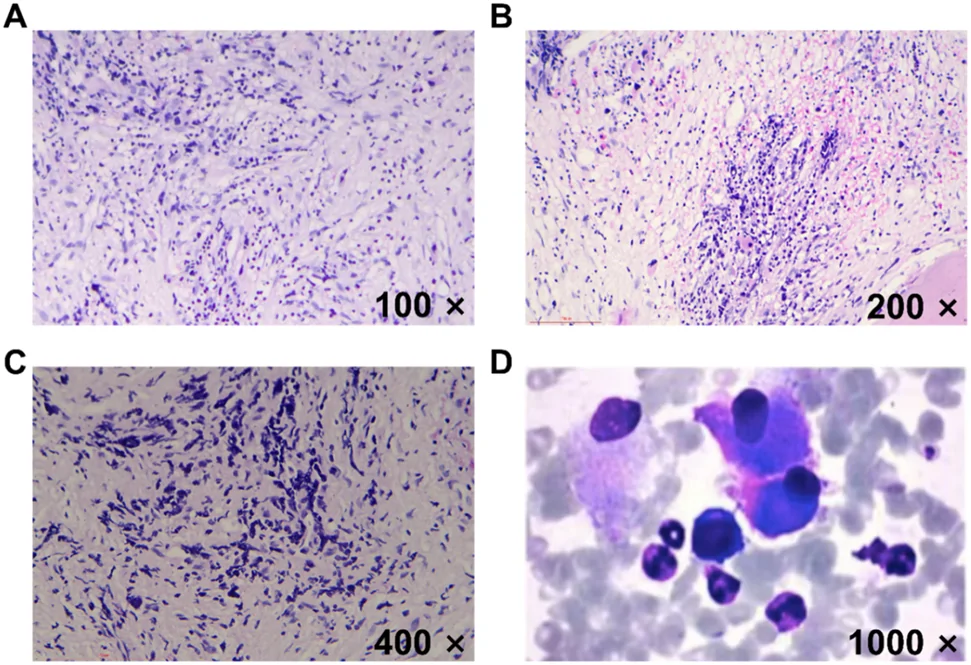
Bone marrow findings. Bone marrow aspirate and biopsy indicated abnormal hematopoiesis with severe reticulin fibrosis. The aspirate revealed left-shifted granulopoiesis, erythroid hypoplasia, severely decreased abundance of megakaryocytes, and reduced PLT counts. Bone marrow biopsy revealed marked reticulin fibrosis consistent with MF-3.

**Figure 4:**
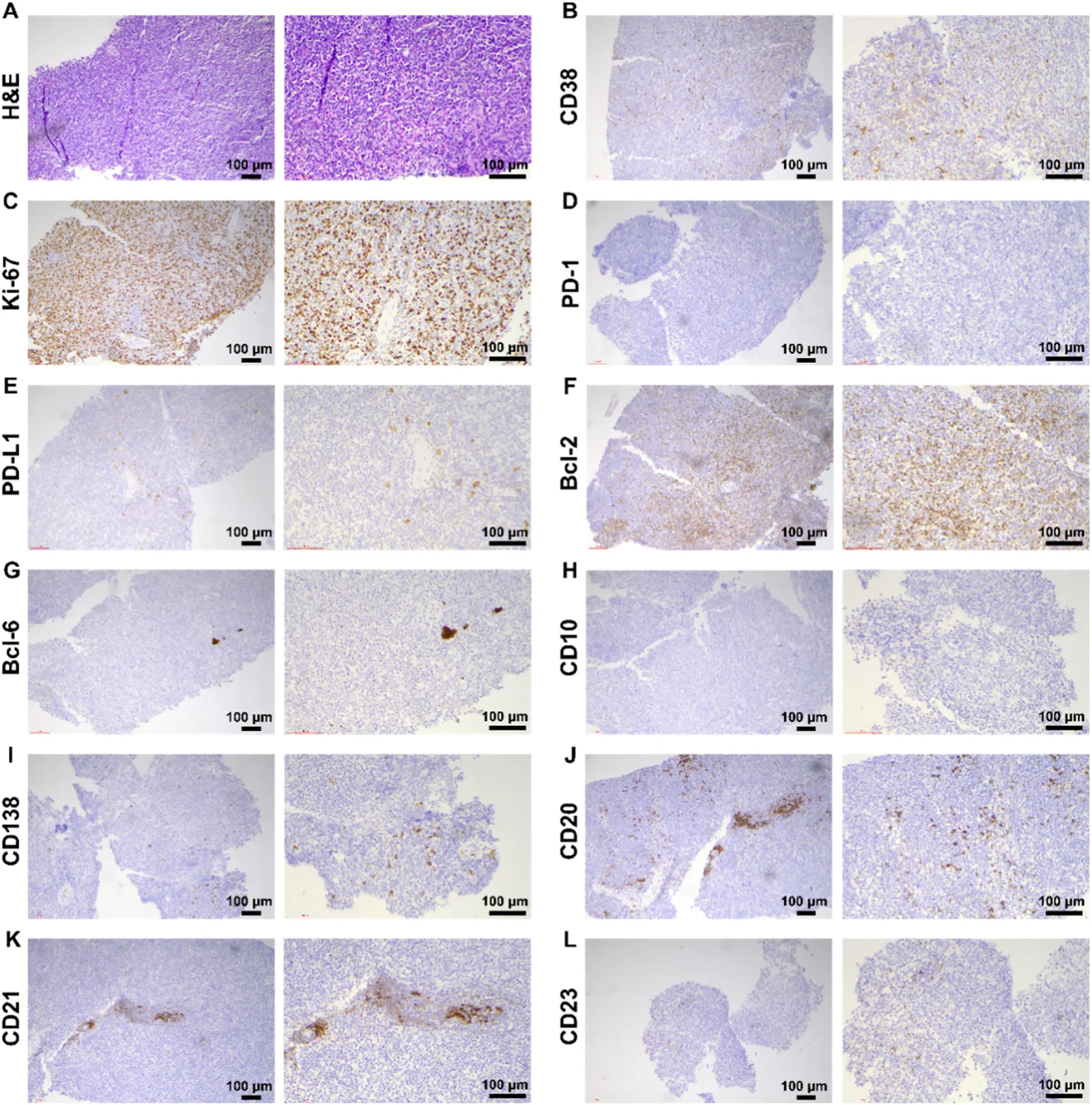
Pathological smear. (A) The lymph node architecture was effaced, and a large number of medium-sized round cells were observed, with some showing atypia. These alterations were associated with interstitial infiltration of neutrophils and eosinophils. These features were consistent with a malignant hematolymphoid neoplasm; therefore, leukemia was highly suspected, (B–L) the results of IHC staining: CD38 (focal +), Ki-67 (55 % +), PD-1 (−), PD-L1 (TPS < 1 %), TdT (scattered +), BCL-2 (+), BCL-6 (−), CD10 (−), CD138 (focal +), CD20 (scattered and focal +), CD21 (FDC +), CD23 (FDC +).

**Figure 5:**
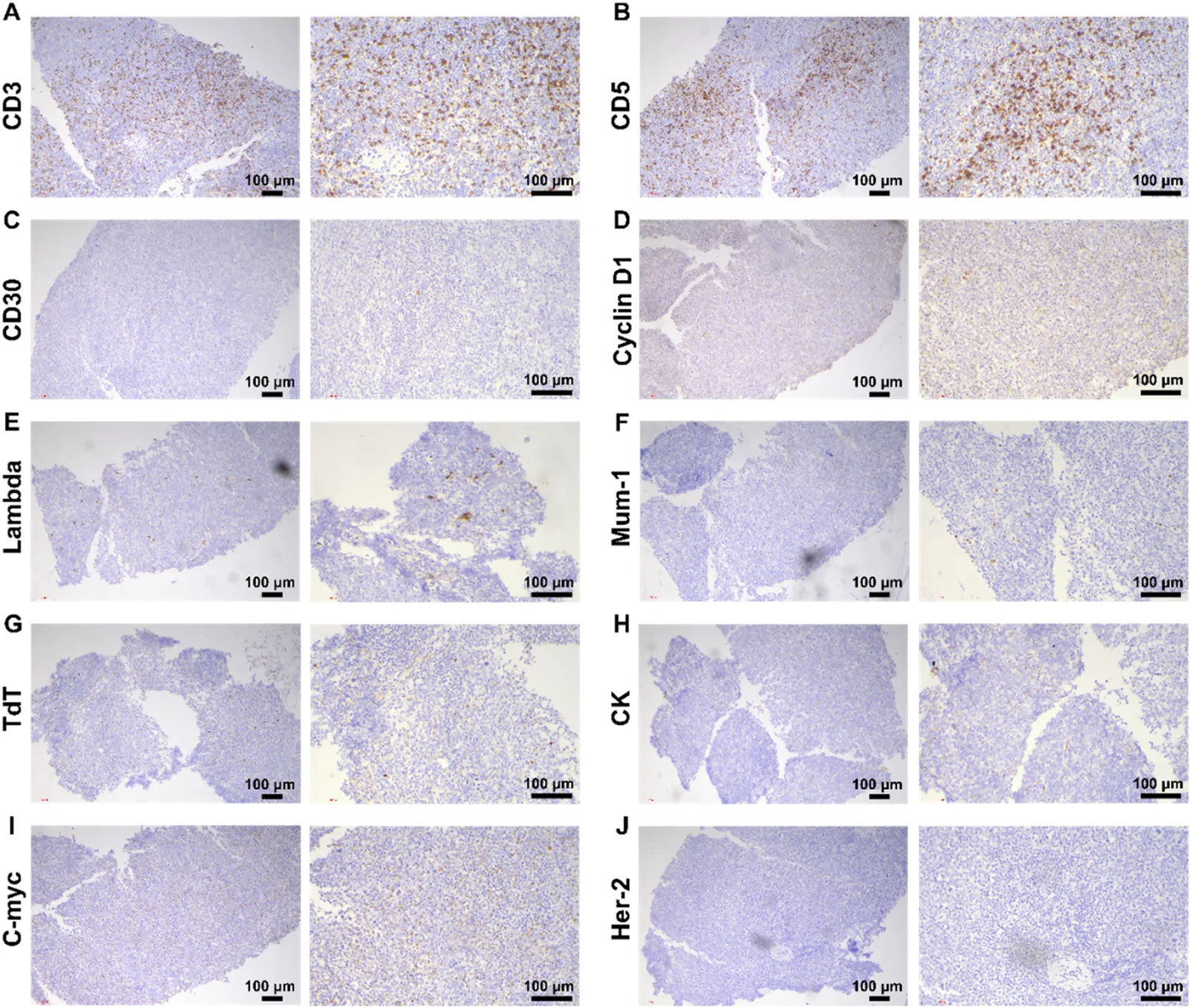
The results of IHC staining. (A–J) CD3 (scattered +), CD5 (scattered +), CD30 (focal +), cyclin D1 (−), lambda (individual +), MUM-1 (individual +), TdT (individual +), CK (−), c-Myc (hotspot: 40 % +), HER-2 (−).

The bone marrow puncture showed a “dry tap” due to severe fibrosis. Subsequent bone marrow biopsy revealed a markedly decreased abundance of hematopoietic cells, accompanied by extensive reticulin fibrosis graded as MF-3 and increased abundance of morphologically abnormal megakaryocytes (Figure 3). Routine screening for genes linked to myeloproliferative neoplasms, including BCR::ABL1, JAK2, MPL, and CALR, was negative. Due to the progressive enlargement of cervical lymph nodes and PET-CT findings, we conducted ultrasound-guided fine-needle aspiration biopsy of the cervical lymph node (Figures 4 and 5). Histopathological examination indicated diffuse infiltration of medium-to-large blast cells into the bone marrow, with immunophenotypic features consistent with myeloid differentiation. Immunohistochemical (IHC) staining confirmed the myeloid origin of these blasts, with strong positivity for myeloperoxidase (MPO), CD117, CD61, and CD235a. Moreover, the Ki-67 proliferation index was as high as 70–75 %. Based on these findings, a diagnosis of myeloid sarcoma was established.

**Figure 6:**
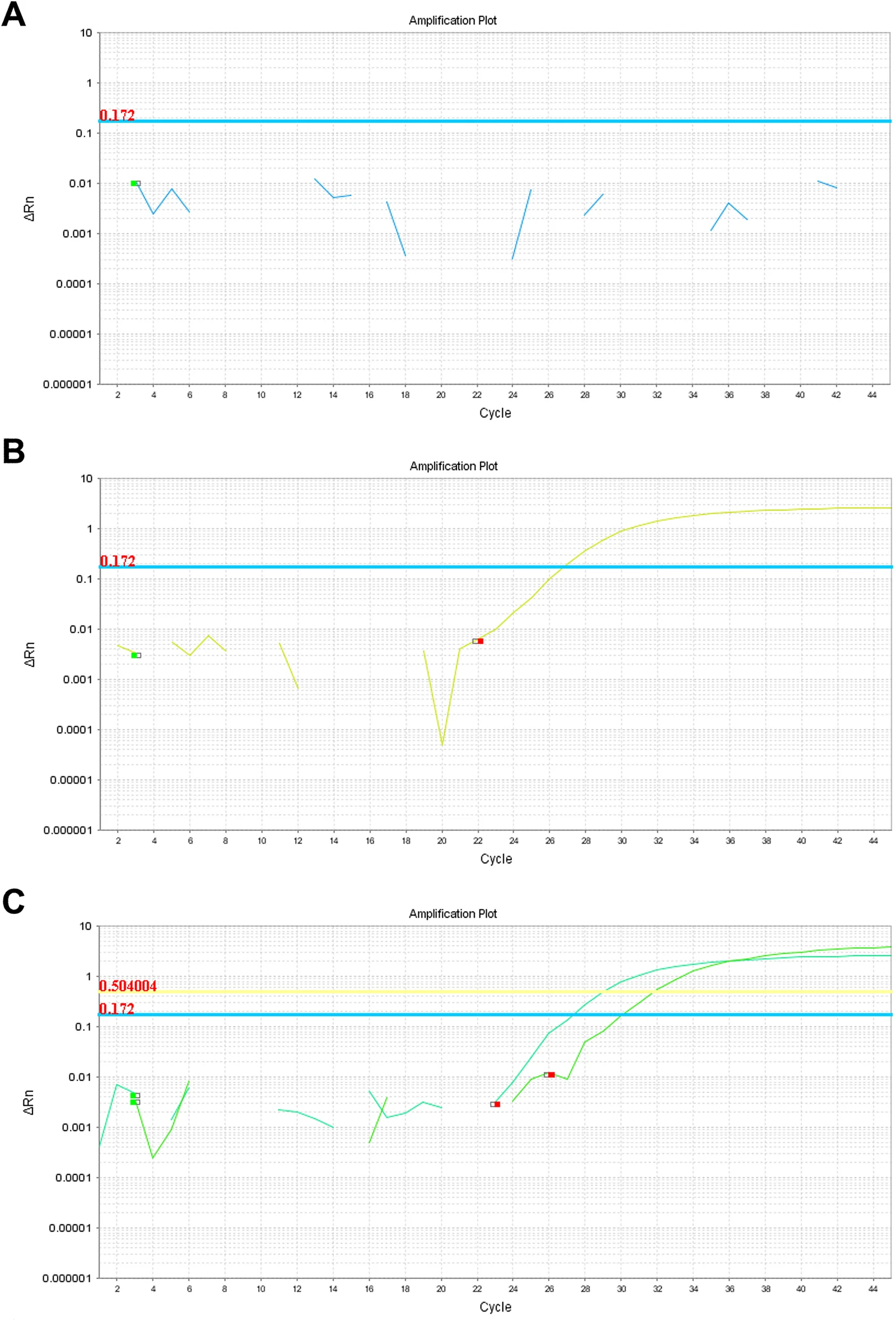
Molecular diagnosis of the rare e6a2 BCR-ABL1 fusion transcript using RT-qPCR. (A) No amplification was observed in the blank control, ruling out system contamination, (B) the positive control showed a standard amplification curve, validating the assay for the e6a2 target, (C) a definitive amplification curve for the e6a2 BCR-ABL1 fusion transcript was detected in the patient’s sample, providing definitive diagnostic evidence for this rare molecular subtype of CML with myeloid sarcoma.

Genetic analysis of bone marrow cells indicated clonal evolution: 47, XY, +8, t(9; 22)(q34; q11.2) [3]/46, XY [11]. Trisomy 8 (+8) occurred in the context of the Ph chromosome, suggesting that the disease was in an advanced stage [12]. RT-qPCR confirmed the rare BCR-ABL1 e6a2 transcript, with a BCR-ABL1/ABL1 ratio of 12.545 %. Besides, the common p210 and p190 transcripts were not detected (Figure 6).

### 2.4 Therapeutic intervention and outcomes

The final diagnosis was BCR-ABL1 e6a2-positive CML in extramedullary blast crisis, accompanied by grade 3 myelofibrosis, active pulmonary tuberculosis, and type 2 diabetes mellitus. After the initial supportive care, including red blood cell transfusion, the patient received TKI-based anti-leukemic therapy and individualized treatment for tuberculosis.

The diagnosis of active pulmonary tuberculosis was established shortly before admission, based on a previous history of tuberculosis, cavitary upper-lobe lesions on chest CT, and a sputum smear positive for acid-fast bacilli. The initial therapeutic regimen for tuberculosis consisted of pasiniazid, rifapentine, pyrazinamide, ethambutol, and levofloxacin. Because of thrombocytopenia and potential drug–drug interactions between rifamycins and anti-leukemic agents, rifapentine was discontinued a few days after initiating the therapeutic regimen for tuberculosis. Thereafter, a rifamycin-sparing regimen consisting of pasiniazid, pyrazinamide, ethambutol, and levofloxacin was continued under the guidance of tuberculosis specialists. Rifampicin and rifapentine were discontinued while receiving dasatinib-, olverembatinib-, and venetoclax-based therapeutic regimens since they are strong inducers of CYP3A4/P-glycoprotein and could reduce the exposure and efficacy of these anti-cancer drugs. After treatment modification, repeated sputum smears and molecular tests for mycobacteria returned negative, with no evidence of uncontrolled tuberculosis during the treatment period.

Dasatinib was combined with the IA chemotherapy regimen consisting of idarubicin and cytarabine. Partial remission (PR) was achieved following two cycles of treatment. Subsequently, olverembatinib combined with the VA regimen, consisting of venetoclax plus azacitidine, was administered for another two cycles. This combination resulted in complete bone marrow remission (CR), and the maximum lymph node diameter was decreased to less than 1 cm. Follow-up PET-CT showed complete metabolic remission. Other abnormal indices also improved (Figure 1). The LDH level declined rapidly, pancytopenia resolved gradually, the size of cervical lymph nodes showed a marked reduction, and repeat bone marrow aspiration revealed active cellularity with fibrosis grade ≤ 1.

At the last follow-up, the patient was receiving maintenance therapy with olverembatinib and venetoclax, and allogeneic hematopoietic stem cell transplantation was planned after achieving adequate control of pulmonary tuberculosis.

## 3 Discussion

This case demonstrates an extremely rare and highly deceptive phenotype of CML in clinical practice. Driven by the rare e6a2 transcript, this phenotype of CML initially presents with extremely severe myelofibrosis and pancytopenia as its “mask”. Extramedullary blast crisis-type myeloid sarcoma is the hallmark clinical feature of this phenotype of CML, and is accompanied by concurrent active pulmonary tuberculosis. This case offers valuable insights into molecular mechanisms, atypical clinical features, and the differential diagnosis of e6a2-positive CML. The vast majority of classic cases of CML (95 %) express the p210 transcript formed by the fusion of BCR exon 13/14 with ABL1 exon 2 [13]. The e6a2 variant is extremely rare globally. Previous studies have suggested that e6a2-positive CML exhibits aggressive clinical behavior, possibly because the earlier BCR breakpoint alters regulatory regions involved in BCR-ABL1 signaling. However, the precise biological mechanisms underlying the aggressive behavior of e6a2-positive CML remain incompletely defined [7].

Clinically, e6a2-positive CML has frequently been reported in the accelerated phase or blast crisis. It may be associated with extramedullary involvement in sites such as lymph nodes, skin, or the central nervous system [14], 15]. In this case, the patient was diagnosed with myeloid sarcoma, and bone marrow cytogenetic analysis revealed additional chromosomal abnormalities (+8) superimposed on the Ph chromosome. Previous studies have indicated that trisomy 8 is one of the most common markers of clonal evolution and disease progression in CML [16]. This case clearly illustrated the highly unstable and rapidly progressive biological behavior of leukemia clones in e6a2-positive CML.

The most prominent diagnostic pitfall in this case was the initial presentation with severe pancytopenia, rather than the marked leukocytosis typically observed in classic CML. Grade 3 myelofibrosis (MF-3) resulted in a “dry tap” on bone marrow aspiration; thus, clinical and morphological features could be easily confused with primary myelofibrosis (PMF) or myelofibrosis associated with myelodysplasia [17]. A few reports have described CML initially presenting with severe myelofibrosis and pancytopenia. Such cases are usually associated with atypical fusion transcripts, such as p190 or e6a2, or advanced disease stages. Extensive reticulin fiber deposition acts like a “cage”, restricting the release of malignant hematopoietic cells into the peripheral blood, thereby masking leukemic blasts and their active proliferation. This case suggests that for any patient with severe myelofibrosis and pancytopenia, CML cannot be excluded even in the absence of classic hematologic or molecular features, especially when molecular tests rule out myelofibrosis. Routine and early molecular screening for atypical BCR-ABL1 fusion transcripts is warranted in such cases. In this case, the presence of t(9;22)(q34;q11.2) despite negative results for common transcripts p210 and p190 prompted further testing for atypical BCR-ABL1 transcripts. Finally, RT-qPCR confirmed the e6a2 fusion.

Another important lesson from this case is the coexistence of active pulmonary tuberculosis and myeloid sarcoma, forming a highly misleading “diagnostic smokescreen”. Clinically, physicians tend to apply Occam’s razor [11], attempting to explain cervical lymphadenopathy and systemic fatigue solely by the already diagnosed tuberculosis. However, patients with malignant tumors are prone to opportunistic infections and tuberculosis reactivation due to immune deficiency [18]. Recent immunological studies have explained that the dysfunctional innate immunity and altered tumor microenvironment in myeloid malignancies can severely impair macrophage function, contributing to the uncontrolled proliferation of *Mycobacterium tuberculosis* [19]. In this dilemma, the case perfectly illustrates Hickam’s dictum [20]. The prompt detection of markedly elevated serum levels of uric acid and LDH, and the detection of the Ph chromosome in the absence of common BCR-ABL1 transcripts have considerably contributed to the diagnosis in this study. Significant increases in the serum levels of LDH and uric acid indicate rapid cellular turnover and impending tumor lysis syndrome, which is extremely rare in uncomplicated focal tuberculosis and strongly suggests a hematologic malignancy [21], 22]. Furthermore, although 18F-FDG PET-CT faces limitations in distinguishing granulomatous infections from malignant tumors because of metabolic overlap, it helped map the distribution of FDG-avid lesions and guided the selection of an accessible lymph node for tissue biopsy in this case. Given the coexistence of active tuberculosis, histopathological confirmation was necessary to determine whether all enlarged lymph nodes were associated with infection.

Due to its rarity, standardized treatment guidelines for e6a2-positive CML are currently lacking. Another clinically important issue in this case was the management of drug-drug interactions between anti-tuberculosis medications and anti-leukemic agents. Rifampicin and rifapentine are potent inducers of CYP3A4 and P-glycoprotein; thus, their co-administration might markedly decrease the plasma exposure of dasatinib and venetoclax. Specifically, these anti-tuberculosis medications are not recommended with olverembatinib because of reduced efficacy in the treatment of leukemia. Therefore, after multidisciplinary discussion with tuberculosis specialists, rifapentine was discontinued, and a rifamycin-sparing regimen containing pasiniazid, pyrazinamide, ethambutol, and levofloxacin was prescribed during treatment with TKI- and venetoclax-based regimens. This strategy allowed simultaneous control of tuberculosis while avoiding major pharmacokinetic alterations that could have compromised the treatment of blast-crisis CML. Previous sporadic case reports have shown that some patients initially respond to first-generation TKIs (imatinib); however, most patients experience treatment resistance and relapse, often due to the intrinsically high kinase activity of e6a2 and frequent clonal evolution. Given the aggressive features of this case, including extramedullary blast crisis, trisomy 8-associated clonal evolution, and severe myelofibrosis, intensive treatment was adopted. The patient was initially treated with the combination of dasatinib and IA chemotherapy, followed by treatment with olverembatinib combined with venetoclax and azacitidine. This therapeutic regimen aligns with the latest advancements in the management of advanced CML. Recent clinical trials have reported that olverembatinib, a novel third-generation TKI, exhibits potent efficacy not only against the T315I mutation but also against heavily pretreated and advanced-phase CML [23]. Furthermore, emerging data indicate that CML blast cells heavily rely on BCL-2 for survival.

The triplet therapy consisting of a potent TKI, a BCL-2 inhibitor (venetoclax), and a hypomethylating agent (azacitidine) has recently shown promising synergistic effects, serving as an effective bridge to transplantation in blast-phase CML [24], 25]. However, while this individualized regimen proved successful in this study, we acknowledge the inherent limitations of a single-case report. The generalizability of the observed treatment efficacy and the long-term outcomes of e6a2-positive CML in extramedullary blast crisis require further validation through larger cohort studies. This sequential TKI-based strategy, together with chemotherapy and individualized anti-tuberculosis therapy, rapidly reduced tumor burden, improved cytopenias, resolved lymphadenopathy, and markedly improved bone marrow fibrosis. Compared with first-generation TKIs, potent BCR-ABL1 inhibitors may be preferable in advanced-phase or high-risk CML, although evidence regarding e6a2-positive CML remains limited to case reports and small series. However, multiple studies have consistently indicated that, regardless of TKI response, early referral for allogeneic hematopoietic stem cell transplantation (allo-HSCT) remains the only curative option for patients with blast crisis or high-risk rare variants of CML [26]. The patient reported in this study achieved hematologic and bone marrow remission, exhibiting complete metabolic remission on PET-CT. Given the high-risk molecular subtype and blast-crisis presentation, allo-HSCT was planned after achieving adequate control of pulmonary tuberculosis.

## 4 Conclusions

BCR-ABL1 e6a2-positive CML is a unique and highly aggressive clinical entity that may present atypically with pancytopenia and severe myelofibrosis. Extramedullary blast crisis (myeloid sarcoma) may be the predominant initial clinical manifestation of BCR-ABL1 e6a2-positive CML. The coexistence of BCR-ABL1 e6a2-positive CML with granulomatous infections, such as tuberculosis, can profoundly increase diagnostic complexity. Comprehensive BCR-ABL1 molecular testing should be conducted as soon as possible for patients with atypical clinical features but evidence of t(9; 22) or Ph chromosome-related abnormalities. This case reaffirms the value of multimodal diagnostic approaches, including metabolic evaluation using PET-CT, detailed histopathological examination, and broad molecular screening. These strategies are critical for identifying rare leukemia variants and facilitating timely, precise targeted intervention.
